# Supplementary material for: MetaRibo-Seq measures translation in microbiomes
Source: Nat Commun. 2020 Jun 29;11:3268. doi: 10.1038/s41467-020-17081-z (PMC7324362; doi:10.1038/s41467-020-17081-z)
Supplement: Supplementary file 10 — Supplementary Data 7 [file 41467_2020_17081_MOESM10_ESM.zip › File2/Confidence_High_Taxonomy/222009_out.krona.html]

Javascript must be enabled to view this page.

members
magnitude
magnitudeUnassigned
count
unassigned
taxon
rank

222009\_out

5

1

SRS1055033\_contig\_number\_contig-100\_28007.28008

2
superkingdom

SRS019029\_contig\_number\_contig-100\_35394.73178
4
1

1
201174
phylum

1
1760
class

2037
order
1

family
2049
1

1
1654
genus

species
712122

SRS013170\_contig\_number\_11539
1

1
phylum
508458

1
class
649775

649776
order
1

1
649777
family

1
genus
1434006

species
651822

SRS144000\_contig\_number\_27103
1

1
phylum
1224

1
class
28211

1
356
order

1
family
69277

1

SRS043018\_contig\_number\_11248
68287
genus
